# Evaluation of a support system for health professionals confined by COVID-19

**DOI:** 10.11606/s1518-8787.2021055003735

**Published:** 2021-12-02

**Authors:** Rosa García-Sierra, Eduard Moreno-Gabriel, Esther Badia Perich, Victòria Sabaté Cintas, Josep M. Bonet Simó, Concepción Fors Violán, Nuria Prat Gil, Mònica Piña Rodríguez, Pere Torán-Monserrat

**Affiliations:** I Unitat de Suport a la Recerca Metropolitana Nord IDIAPJGol Mataró Barcelona España Unitat de Suport a la Recerca Metropolitana Nord. Fundació Institut Universitari per a la Recerca a l’Atenció Primària de Salut Jordi Gol i Gurina (IDIAPJGol). Mataró, Barcelona, España; II Universitat Autònoma de Barcelona Facultad de Medicina Departamento de Enfermería Barcelona España Universitat Autònoma de Barcelona. Facultad de Medicina. Departamento de Enfermería. Barcelona, España; III Grup de Recerca Multidisciplinar en Salut i Societat Barcelona España Grup de Recerca Multidisciplinar en Salut i Societat (GREMSAS; 2017 SGR 917). Barcelona, España; IV Àrea de Qualitat i Seguretat del Pacient Direcció d’Atenció Primària Metropolitana Nord Institut Català de la Salut Sabadell Barcelona España Àrea de Qualitat i Seguretat del Pacient. Direcció d’Atenció Primària Metropolitana Nord. Institut Català de la Salut. Sabadell, Barcelona, España; V Direcció d’Atenció Primària Metropolitana Nord Institut Català de la Salut Sabadell Barcelona España Direcció d’Atenció Primària Metropolitana Nord. Institut Català de la Salut. Sabadell, Barcelona, España; VI Unitat Bàsica de Prevenció Institut Català de la Salut Masnou Barcelona España Unitat Bàsica de Prevenció. Gerència Territorial Metropolitana Nord. Institut Català de la Salut, Masnou, Barcelona, España; VII Universitat de Girona Facultad de Medicina Departmento de Medicina Girona España Universitat de Girona. Facultad de Medicina. Departmento de Medicina. Girona, España

**Keywords:** Health Personnel, psychology, Self-Help Groups, Telephone, Physical Distancing, COVID-19, Health Impact Assessment

## Abstract

**OBJECTIVE:**

To evaluate the implementation of a telephone system in a department of Primary Care in Barcelona, Spain, supporting health professionals confined by COVID-19.

**METHODS:**

We conducted an observational, descriptive, cross-sectional study with confined professionals, between March 11 and May 31, 2020. We emailed a questionnaire with 18 closed-ended questions and one open-ended question and performed a descriptive analysis of the closed-ended answers and an analysis of the thematic content of the open-ended question.

**RESULTS:**

Thirty-nine hundred and ninety-eight professionals evaluated the system overall with a score of 6.54 on a scale of 1 to 10. The evaluation of the format of calls made in the support system had higher scores, while the psychological support unit and the coordination of the different groups had lower scores. The content analysis of the open-ended question provides explanatory arguments for the quantitative results.

**CONCLUSIONS:**

The study allowed a valid and reliable evaluation of the implementation of a support system for confined professionals, in addition to recognizing areas for improvement.

## INTRODUCTION

COVID-19 is an emerging disease that, as of March 18, 2021, has globally affected 120 million people and has already caused 2.6 million deaths since the start of the pandemic^1^.

Health care workers were the first line of response to the outbreak, exposed to unprecedented risk to themselves and their families. According to the World Health Organization (WHO), data from many countries indicate that the number of SARS-CoV-2 infections among healthcare workers is much higher than among the general population, and thousands of healthcare workers infected with SARS-CoV-2 have lost their lives worldwide^2^.

The latest report on healthcare professionals in Spain, published by the National Epidemiological Surveillance Network (*Red Nacional de Vigilancia Epidemiológica*) on May 21, 2020, reported 40,921 cases of COVID-19^3^. Subsequent reports on the overall situation in the country show that the disease in healthcare professionals has continued to appear, with 82,690 cases reported as of July 14, 2021^4^. However, seroprevalence data point to higher prevalences in healthcare personnel: 10.3% of a sample of primary care and hospital professionals in the northern metropolitan region of Barcelona were positive for anti-SARS-CoV-2 IgG, with no differences observed between primary care and hospital personnel^5^.

As the 2003 SARS-1 outbreak showed, healthcare professionals are exposed to the virus and to numerous stressors, such as high workloads, stress, fatigue, exhaustion and stigma. In addition to this set of threats, domestic isolation of suspected SARS-CoV-2 cases is one of the main non-pharmacological interventions to mitigate and even suppress its spread, constituting a predictor of post-traumatic stress disorders^6^.

Faced with the first appearance of a contact by a healthcare professional with a patient positive for SARS-CoV-2 and the consequent prescription for isolation, the administration of the North Metropolitan Primary Care Area of Barcelona (Spain) –healthcare area that provides service to 1,448,812 citizens from 70 municipalities in the province of Barcelona, and which has a staff of more than 4,500 professionals– launched a telephone service system to all professionals who were prescribed a home confinement between the February 17 and May 31, 2020, the period corresponding to the first wave of the epidemic.

Considering the information available at that time on the evolution of the disease, the system was provided with a “biopsychosocial” orientation, since it intended to follow-up the clinical evolution of the professionals who presented symptoms at some point in the follow-up, in addition to offering recommendations and support in psychosocial issues related to the circumstances of the isolation, whether symptoms had developed or if the isolation was due to contact with positive patients. The follow-up team was interdisciplinary, made up of 16 nursing professionals, 5 family medicine professionals, 1 psychiatrist, 5 psychologists, and 1 professional from social work, under the administration of the Prevention Unit, responsible for Occupational Health of the organization. The follow-up consisted of telephone calls to follow-up the progress of the symptoms, assessment and response to psychosocial needs that may appear. The follow-up team met weekly to guarantee criteria for action and management of specific cases. The periodicity determined for making the calls was 72 hours, although it was a decision of the professionals to increase the time of calls, with a total of 10,419. The descriptive analysis of the sociodemographic and clinical characteristics of the professionals assisted in this support system was published recently^9^.

The objective of this research was to know how the professionals who had contact with positive patients or who manifested symptoms of COVID-19 evaluated both the care received and the functioning of the biopsychosocial system, as well as identify the needs perceived as poorly attended, with a view to implementing improvements in the system, in case of future needs.

## METHODS

### Design

Observational, descriptive and transversal study, carried out with the workers of the administration of Primary Care of the North Metropolitan, in Barcelona, Spain. Inclusion criteria were to have confined at home by symptomatology or contact with COVID-19 between March 11 and May 31, 2020, and to have provided at least one contact email during the period of confinement. We did not define any exclusion criteria and we invited 1,247 professionals who met the inclusion criteria to participate, 398 responded our inviting, representing a response rate of 32%.

### Data Collection

The data were collected by the Research Support Unit of the North Metropolitan Area of the Catalan Health Institute (ICS - *Instituto Catalán de la Salud*), a department that provides professional and logistical support to organize research initiatives that may arise in the territory. We distributed a questionnaire by e-mail through a survey management platform (Box).

**Box:** Assessment questionnaire of the support system for health professionals confined by COVID-19.

Assessment questionnaire of the support system for health professionals confined by COVID-19
As a professional affected by COVID-19 did you receive any telephone call follow-up by this system while you were confined?
Answer YES: Start the survey Answer NO: Direct to suggestions
Evaluate from 0 (not satisfactory) to 3 (very satisfactory) the following aspects of the follow-up calls:
1) Adequate frequency of clinical follow-up calls.
2) Duration of telephone calls.
3) Information received in the calls.
4) Resolution of the doubts raised during the calls.
5) Support received during clinical follow-up.
Evaluate from 0 (not useful) to 3 (very useful) the extent to which the calls was useful for you in the following aspects:
6) Emotional well-being.
7) Management related to labor issues.
8) Family relationships.
9) Symptom management.
Evaluate from 0 (not satisfactory) to 3 (very satisfactory) the task performed by the following groups involved in the system:
10) The professionals who called you for clinical follow-up.
11) Psychological support unit.
12) Basic Prevention Unit.
13) PCR performing system.
14) Management team of your center.
15) Support from the Health Area Management.
16) Coordination between the different agents involved.
17) Facility to contact the professionals who carried out the follow-up, in case it was necessary.
18) If you had to give a grade to the follow-up service in general, what grade would you give it from 1 to 10 (1 = very bad, 10 = very good)?
19) Do you have any suggestions for improvement or comments?

This questionnaire made it possible to obtain information on the previously defined study variables. We conducted the surveys during September 2020.

### Instruments

We developed an *ad hoc* questionnaire following the recommendations of Boynton and Greenhalgh^10^ to ensure the validity and reliability of the questionnaires created. The Quality Department requested the research and the first step was to find out what issues they wanted to explore. Then, we determined the most appropriate methodology for data collection, since we were dealing with the quantification of general measures, and seeking ease and speed in filling out, we opted for a 17-question questionnaire with a numerical scale from 0 to 3, and one question with an answer from 1 to 10. In order to expand on the findings, the participants had the possibility to answer an open-ended question, which was analyzed using qualitative methods.

We considered not advisable to use a standardized measuring instrument due to the particular context of the pandemic experienced for the first time in a specific health care setting, for which any questionnaire used in another context would lose its validity.

In relation to the appearance of the questionnaire, we took care of the details related to font, font size, and length of the questions to ensure a high response rate. In addition, we conducted 5 pretest cognitive interviews with a sample of potential study participants to evaluate a first version of the questionnaire using the think aloud technique^11^.

We ensured the validity of the questionnaire using a procedure giving specific instructions to 6 expert judges asked to evaluate the Interpretability, Representativeness, Relevance of the items, by scoring from 1 to 4 for each item, after previous information about the purpose of the questionnaire. We also encouraged experts to share comments on writing of items and response options, suggestions for revisions, number and order of items, instructional text, missing items, headlines and layout^12^. The Content Validity Index for the final closed-response items (I-IVC) with 6 expert judges was greater than 0.78 for all items. Cronbach’s α for the closed-response items was 0.93.

### Data Analysis

We performed a descriptive analysis of the quantitative data presenting the categorical variables with frequency distribution, and the quantitative variables with mean, standard deviation, mode and median. We used Student’s t-test and ANOVA for the comparison of means, and any value of p < 0.05 was considered significant. We used the SPSS package, version 25.

Using the ATLAS.ti 8.4 program, the answers to the open-ended questions were subjected to a summative thematic content analysis, which involves coding and counting the answers based on a pre-established matrix of codes and interpreting the latent meaning of this content based on the contexts of use and co-occurrences of the codes^13^. We conducted the quantitative and qualitative analyses in a sequential and explanatory manner, orienting the qualitative analysis to understanding of the quantitative results.

### Ethical Considerations

The first question of the questionnaire involved informed consent, if the participant accepted it, it was possible to continue the survey. All participants were volunteers, with anonymity guaranteed by deactivating the possibility of tracking answers, and no request for data that could reveal the participant’s identity. The Clinical Research Ethics Committee of IDIAPJGol (Code 20/209-PCV) approved the study.

## RESULTS

We sent the survey link to 1,247 people, of whom 398 initiated the survey. In the first question, 60 participants answered that they did not recall receiving the evaluated support, so the survey directed them to the open-ended comments and suggestions question, and 22 of them wrote comments on it. Of the remaining 338 participants who did recall receiving telephone support, 286 completed the entire survey.

### Sociodemographic Data

The sample was predominantly female (81.8%). The mean age was 47 years with a standard deviation of 10.4, minimum age 22 years and maximum age 65 years. Other sociodemographic characteristics are in Table 1.

**Table 1:** Sociodemographic characteristics.

n = 286	Yes	No
Belongs to a vulnerable group	88 (30.8%)	198 (69.2%)
Lives with vulnerable people	98 (34.3%)	188 (65.7%)
Lives with children	166 (41.9%)	120 (58%)
Was diagnosed with COVID-19 by PCR	127 (44.4%)	159 (55.6%)
Knows the origin of the infection	63 (46%)	74 (54%)

PCR: polymerase chain reaction.

Nurses and physicians were up to 63% of those responding the questionnaire and other groups of professionals were the rest of the sample. Table 2 shows the distribution of the participants according to their profession.

**Table 2:** Distribution of the sample according to profession (n = 286).

Profession	n (%)
Nurse	102 (35.7%)
Physician	80 (28%)
Management and services	49 (17.1%)
Auxiliary nursing care technician	24 (8.4%)
Social Work	7 (2.4%)
Odontology	6 (2.1%)
Management Technician	4 (1.4%)
Midwife	2 (0.7%)
Physiotherapist	1 (0.3%)
Other professions	11 (3.8%)

### Quantitative Evaluation Results

The mean overall score for the system was 6.54, with the worst possible score being 0 and the best possible score being 10. The mode was 8, and the median was 7. Table 3 shows the score obtained for each of the items on a scale of 0 to 3, showing that the highest scores correspond to the items related to the formal aspects of the calls, and the lowest score corresponds to the psychological support unit.

**Table 3:** Evaluation of the system.

Aspects related to calls	Mean^a^	SD	Mode^a^
Adequate frequency	2.11	0.89	3
Duration of calls	2.37	0.73	3
Information received	2.16	0.86	3
Resolution of doubts	2.11	0.88	3
Support received	2.15	0.93	3
Usefulness of the calls			
Emotional well-being	1.91	0.98	3
Work management	1.88	1.02	3
Family relationships	1.73	1.06	2
Symptom management	2.00	0.96	3
Valuation by group			
Identification	2.76	0.56	3
Follow-up professionals	2.34	0.78	3
Psychological Support Unit	0.70	1.11	0
Basic Prevention Unit	1.64	1.21	3
PCR implementation system	1.66	1.18	3
Center management team	2.15	1.02	3
Support from the general management	1.44	1.12	0
Coordination and management			
Coordination of groups	1.47	0.95	1
Ease of contact	1.61	1.10	1/3

SD: standard deviation.

^a^ 0 is the lowest possible score and 3 is the highest possible score.

We performed a T test to compare the overall mean obtained according to gender, relevance to a vulnerable group, cohabitation with vulnerable persons, having children or not, PCR result, being a health sciences professional or not, and origin of infection. We did not find significant differences in any of the cases. Table 4 shows the statistics of these mean comparisons.

**Table 4:** Comparison of overall score by group.

	Mean	SD	t^a^	Sig^c^
Sex				
Man	6.88	2.49	1.11	0.27
Woman	6.47	2.41		
Belongs to vulnerable groups				
No	6.58	2.36	0.37	0.71
Yes	6.47	2.59		
Living with vulnerable people				
No	6.48	2.35	-0.64	0.52
Yes	6.67	2.57		
Sons/daughters				
No	6.77	2.36	1.81	0.07
Yes	6.24	2.49		
PCR result				
Negative	6.50	2.50	-0.80	0.42
Positive	6.74	2.34		
Health sciences profession				
No	6.82	2.28	1.11	0.27
Yes	6.45	2.47		
Origin of infection			F^b^	
Patient	6.45	2.38	0.85	0.43
Community	7.42	2.11		
Unknown	6.74	2.38		

SD: standard deviation.

^a^ Student t-test.

^b^ F Test.

^c^ Service and management.

We analyzed in more detail the result obtained in the evaluation by the psychological support unit, since it obtained a very low score. We performed bivariate analyses with all sociodemographic and occupational variables, finding no significant association.

### Results of the Content Analysis of the Answers to the Open-Ended Question

A total of 109 people (27.4%) answered the question “If you have any suggestions for improvement or comments, you can write them here” formulated to all participants.

Analysis of the manifest content of the answers, coded according to the three dimensions of the bio-psycho-social approach, showed that most of the comments (n = 61) referred to organizational aspects (social dimension), followed by those referring to psychological issues (psychological dimension; n = 22) and, finally, to issues related to symptom management (biological dimension; n = 10).

### Social dimension

Despite the deductive approach of the analysis, categorizing the data in the three dimensions of the biopsychosocial orientation, the analysis of the context in which the comments were made and their co-occurrences showed how comments referring to the more psychological experience (“I felt very lonely”) and to the biological process of the disease (“I had a fever for 11 days”) were linked to explanations of concrete actions or omissions at the level of the socio-organizational management of the situation (“to demand the performance of the PCR”):

*During my illness I felt very lonely, I had to call to request a PCR, both at the beginning of my illness and in the pre-incorporation period. I had a fever for 11 days and did not receive a follow-up call, I was the one who decided to have a Chest X-ray, manage it, check it and see that there was pneumonia, talk to an internist, who prescribed medication. I did not feel supported at any time. I would not want to repeat an experience like this again. (43:2)*

As in this comment, the professionals located their experience of isolation in one place or another on the accompaniment-loneliness continuum as the effect of a combination of various concrete practices, often linked to the resolution of formal aspects, which may or may not have taken place during follow-up (for example, the performance of diagnostic tests).

### Psychological dimension

*In the first part of the process, my perception was of total abandonment, including the lack of PCR, until I was admitted to the hospital with pneumonia 15 days after the beginning of my confinement. After discharge, yes, I was very comforted by the calls from Dr. M.. She was very supportive psychologically and emotionally. I am very grateful to her. To the UPR, I give it a zero. (88:3)*

This commentary shows the link between the perception of loneliness and some of the weaknesses of the system, especially at the beginning (the performance of PCR and the role of the Basic Prevention Unit –”UPR”, in this quote). However, this loneliness is made up for the role of the psychological support. Dr. M” belongs to the Psychological Support Unit, activated in specific cases. There are few explicit references to “psychological support” (n = 5) in the set of open-ended responses and all, except this expression of gratitude, they are explicit requests to expand or integrate (n = 4) this type of service.

Therefore, it is pertinent to make a distinction between the assessments of the psychological support unit and the perception of feeling emotionally accompanied as a result of received care in the framework of this follow-up system.

### Biological dimension

As noted above, the value of the system as “emotional support” cannot be attributed exclusively to the performance of the Psychological Support Unit, but seems to emerge rather as the effect of a combination of various concrete practices that did or did not take place during follow-up, including those related to symptom management and “self-management of the disease” as detailed in the following quote.

*I consider the follow-up as essential, because of the support on the symptoms and uncertainties, especially at the beginning of the pandemic, when I was infected. About my process, I would also highlight the emotional support I received, I did not expect it and I found it and I was grateful for it. We are “patients” but at the same time health professionals, something that helps to self-manage the disease but, at the same time, to make some anticipations. (106:4)*

## DISCUSSION

Process and outcome indicators of healthcare services play a crucial role in patient satisfaction^14^. The intervention implemented would be along the lines of a Walkround, a common practice associated with patient safety, in which feedback is a powerful intervention^15^.

This research highlights the responsiveness of the healthcare system to a crisis caused by the COVID-19 pandemic that placed the healthcare system in a situation of unprecedented stress, and which had to give a response to healthcare professionals in their role as patients. The professionals affected evaluated the response as satisfactory, giving the intervention an overall score of 6.54.

We designed the evaluation meticulously to ensure that the questions did not contain interpretation biases and that the contents were those really intended to be evaluated. The analysis of the data, with no predictor variable for the results, shows that the participants responded to the questionnaire leaving aside the social and emotional situations that could affect them. Finally, the characteristics of the participants were very similar to those of the total number of confined professionals, as shown by the mean age of 47 years (45 in the total number of confined professionals) and the high percentage of women, 81.8% (78.8% in the total number of confined professionals)^9^.

The formal aspects of the calls had positive evaluation, with a high categorization of the responses.

The biopsychosocial vocation is an important, and achieved, aspect in the implementation of the system, since the scores in the “usefulness of the calls” section are on a high position. The qualitative results show that there are different conceptions of the usefulness that this type of follow-up should have and that the system loses its value when there is no appreciation of this triple aspect.

Many hypotheses can explain this effect in light of the results and related literature. For example, if the preponderance is on the labor aspects, the perception may be balanced towards an intervention of pressure and control of human resources, and not of support^16^. On the other hand, health professionals may not accept a more clinically focused system very well, assuming that they have a certain expertise in this field. Similarly, strictly psychological follow-up would cause rejection in a group where, paradoxically, occur a considerable stigmatization of mental health problems^17^. It is important to consider that the literature shows a considerable proportion of healthcare workers having experienced sleep and mood disorders during the pandemic^18^. Moral damage and the development of mental illness are real risks when working in unprecedented scenarios such as this one. Then, this study emphasizes we must work proactively to prevent the psychological consequences of the pandemic on healthcare workers from becoming severe^19^.

The evaluation of the different groups participating in the system showed heterogeneous scores, including the very low score for the Psychological Support Unit, for which the quantitative analysis was unable to find any predictors to explain it. However, the results of the analysis of the open-ended responses allow us to develop some plausible explanations. On the one hand, the work of the Psychological Support Unit does not receive a negative evaluation, but critics for not having more presence. Data show clearly that the perception of emotional support or accompaniment goes beyond the work of this Unit and emerges as a result of a sum of small actions, often linked to management, information and coordination, facilitating personalized and more empathetic attention.

The score obtained for the coordination of the different groups involved is lower than the midpoint. As expressed in the participants’ comments, the situation makes them tolerant towards this lack of coordination and the difficulty of implementing any initiative, which may result in benevolent scores.

The evidence obtained allows the elaboration of an explanation that turns the unknown into the known, built from a process focused on options, experience and errors, areas that intervene in the process of problem recognition in its early stages of development, the first principle of high reliability organizations^20^.

Given the evidence of possible mental health effects on health professionals during the pandemic, various institutions and organizations have implemented strategies to address these types of problems^21^. An intervention based on mobile devices was implemented in Spain, aimed at preventing and mitigating the most common problems in this situation^22^. The results of this specific evaluation did not show a significant impact of the intervention, so it is particularly relevant to complement this type of evidence with the assessments made by the target groups.

There are some limitations in this study due to the low response rate, and although the reasons for the non-participation of the rest of the people invited to participate are unknown, the time lapse between the confinement period and the conclusion of the research may explain this. However, the heterogeneity of the sample characteristics and of the open-ended question comments do not suggest a selection bias.

In conclusion, we evaluated the implementation of a biopsychosocial support system for primary care health professionals affected by COVID-19 applied in the early stages of the pandemic. The professionals have perceived the system as useful, since the number of scores is in the high range of the scale; however, they recognize some aspects needing improvement. There was variability in the responses which was due to the different expectations of professionals, the different adaptations that occurred as knowledge about the virus increased, and the understanding of the difficulty of the scenario where they were all working.
