# Supplementary material for: Evaluation of a support system for health professionals confined by COVID-19
Source: Rev Saude Publica. 2021 Dec 2;55:108. doi: 10.11606/s1518-8787.2021055003735 (PMC8664052; doi:10.11606/s1518-8787.2021055003735)
Supplement: Translation

# Translation: Evaluación de un circuito de apoyo a profesionales sanitarios confinados por COVID-19

**Authors:** Rosa García-Sierra, Eduard Moreno-Gabriel, Esther Badia Perich, Victòria Sabaté Cintas, Josep M. Bonet Simó, Concepción Fors Violán, Nuria Prat Gil, Mònica Piña Rodríguez, Pere Torán-Monserrat

**Affiliations:** I España Unitat de Suport a la Recerca Metropolitana Nord. Fundació Institut Universitari per a la Recerca a l’Atenció Primària de Salut Jordi Gol i Gurina (IDIAPJGol), Mataró, Barcelona, España; II España Universitat Autònoma de Barcelona. Facultad de Medicina. Departamento de Enfermería. Barcelona, España; III España Grup de Recerca Multidisciplinar en Salut i Societat (GREMSAS; 2017 SGR 917). Barcelona, España; IV España Àrea de Qualitat i Seguretat del Pacient. Direcció d’Atenció Primària Metropolitana Nord. Institut Català de la Salut. Sabadell, Barcelona, España; V España Direcció d’Atenció Primària Metropolitana Nord. Institut Català de la Salut. Sabadell, Barcelona, España; VI España Unitat Bàsica de Prevenció. Gerència Territorial Metropolitana Nord. Institut Català de la Salut, Masnou, Barcelona, España; VII España Universitat de Girona. Facultad de Medicina. Departmento de Medicina. Girona, España

**Keywords:** Personal de Salud, psicología, Teléfono, Distanciamiento Físico, COVID-19, Evaluación del Impacto en la Salud

## Abstract

**OBJETIVOS:**

Evaluar la implementación de un circuito telefónico de apoyo a profesionales sanitarios confinados por COVID-19 en una dirección de Atención Primaria de Barcelona, en España.

**MÉTODOS:**

Estudio observacional, descriptivo y transversal, realizado con profesionales confinados en domicilio entre el 11 de marzo y el 31 de mayo de 2020. Se envió por correo electrónico un cuestionario con 18 preguntas cerradas y una abierta. Se realizó un análisis descriptivo de las respuestas cerradas y un análisis del contenido temático de la pregunta abierta.

**RESULTADOS:**

398 profesionales puntuaron globalmente el circuito con 6,54 en una escala de 1 a 10. El formato de las llamadas realizadas en el circuito de apoyo se estimó con las puntuaciones máximas, la unidad de apoyo psicológico y la coordinación por diferentes colectivos se evaluaron con las puntuaciones más bajas. El análisis del contenido de la pregunta abierta ofrece argumentos explicativos de los resultados cuantitativos.

**CONCLUSIONES:**

El estudio permitió evaluar de manera válida y fiable la implementación de un circuito de apoyo a profesionales confinados, además de reconocer áreas de mejora.

## INTRODUCCIÓN

La COVID-19 es una enfermedad emergente que, el 18 de marzo de 2021, ha afectado globalmente a 120 millones de personas y ya ha causado 2,6 millones de muertes desde el inicio de la pandemia^1^.

Los trabajadores sanitarios constituyeron la primera línea de respuesta al brote y, por lo tanto, estuvieron expuestos tanto ellos como sus familias a un riesgo sin precedentes. De acuerdo con la Organización Mundial de la Salud (OMS), los datos de muchos países indican que el número de infecciones por el SARS-CoV-2 entre los trabajadores de la salud es mucho mayor que entre la población general y miles de trabajadores sanitarios infectados por este virus han perdido la vida en todo el mundo^2^.

El último informe sobre profesionales sanitarios en España, publicado por la Red Nacional de Vigilancia Epidemiológica el 21 de mayo de 2020, notificaba 40.921 casos de la COVID-19^3^. Los informes posteriores sobre la situación general en el país muestran que la enfermedad en personal sanitario ha seguido apareciendo, habiéndose notificado, el 14 de julio de 2021, 82.690 casos^4^. Sin embargo, los datos de seroprevalencia apuntan a prevalencias mayores en personal sanitario: un 10,3% de una muestra de profesionales de atención primaria y hospitalaria de la región Metropolitana norte de Barcelona fue positiva para anti-SARS-CoV-2 IgG, sin observarse diferencias entre el personal de primaria y el hospitalario^5^.

Asimismo, tal y como mostró el brote de SARS-1 de 2003, los profesionales de la salud están expuestos no solo al virus, sino también a numerosos elementos estresantes, como, por ejemplo, elevadas cargas de trabajo, estrés, fatiga, agotamiento y estigma. A este conjunto de amenazas se añade el hecho de que el aislamiento doméstico de casos sospechosos de SARS-CoV-2 y sus contactos sea una de las principales intervenciones no farmacológicas para mitigar e incluso suprimir su expansión, constituyendo un elemento predictor de trastornos por estrés postraumático^6^.

Ante la primera aparición de un contacto de un profesional sanitario con un paciente positivo en SARS-CoV-2 y la consiguiente prescripción de su aislamiento, la dirección de Atención Primaria del área sanitaria Metropolitana Nord de Barcelona (España) –área sanitaria que presta servicio a 1.448.812 ciudadanos de 70 municipios de la provincia de Barcelona, y que cuenta con una plantilla de más de 4.500 profesionales– puso en marcha un circuito de atención telefónica dirigido a todos los profesionales a los que les fue prescrito un confinamiento domiciliario entre el 17 de febrero y el 31 de mayo de 2020, período correspondiente a la primera ola de la epidemia.

Considerando la información disponible en aquel momento sobre la evolución de la enfermedad, se dotó al circuito de una orientación “biopsicosocial”, ya que pretendía hacer un seguimiento de la evolución clínica de los profesionales que presentaban síntomas en algún momento del seguimiento, además de ofrecer recomendaciones y apoyo en cuestiones psicosociales relacionadas con las circunstancias del aislamiento, tanto si se habían desarrollado síntomas como si el aislamiento era por contacto con pacientes positivos. El equipo de seguimiento era interdisciplinar, formado por 16 profesionales de enfermería, 5 de medicina de familia, 1 de psiquiatría, 5 de psicología, y 1 de trabajo social, bajo la dirección de la Unidad de Prevención, responsable de la Salud Laboral de la organización. El seguimiento consistió en llamadas telefónicas para seguimiento del avance de la sintomatología, valoración y respuesta a necesidades psicosociales que pudieran aparecer. El equipo de seguimiento se reunía semanalmente para garantizar criterios de actuación y gestión de casos particulares. La periodicidad que se determinó para la realización de las llamadas fue de 72 horas, aunque quedaba a criterio de los profesionales aumentar la cadencia de llamadas, siendo contabilizadas un total de 10.419. El análisis descriptivo de las características sociodemográficas y clínicas de los profesionales atendidos en este circuito de apoyo se ha publicado recientemente^9^.

Ante esto, el objetivo de esta investigación fue conocer cómo los profesionales que tuvieron contacto con pacientes positivos o que manifestaron síntomas de la COVID-19 evaluaron tanto la atención recibida como el funcionamiento del circuito biopsicosocial, así como identificar cuáles fueron las necesidades que se percibieron como deficientemente atendidas, para poder implementar mejoras en el circuito, en caso de que este volviera a ser necesario.

## MÉTODOS

### Diseño

Estudio observacional, descriptivo y transversal, realizado con los trabajadores de la Dirección de Atención Primaria Metropolitana Nord, en Barcelona, España. El criterio de inclusión fue haber sido confinado en domicilio por sintomatología o contacto con la COVID-19 entre el 11 de marzo y el 31 de mayo de 2020 y haber facilitado al menos un email de contacto durante el período de confinamiento. No se definió ningún criterio de exclusión. Se invitó a participar a los 1.247 profesionales que cumplían los criterios de inclusión, de los que respondieron 398, lo que supone una tasa de respuesta del 32%.

### Recogida de Datos

Los datos fueron recogidos por la Unitat de Suport a la Recerca de la Metropolitana Nord del Instituto Catalán de la Salud (ICS), departamento que brinda apoyo profesional y logístico para organizar las iniciativas de investigación que puedan surgir en el territorio. Se distribuyó un cuestionario por correo electrónico a través de una plataforma de gestión de encuestas (Quadro). Este cuestionario permitió obtener la información de las variables de estudio definidas previamente. Las encuestas se realizaron durante el mes de septiembre de 2020.

**Quadro:** Cuestionario de evaluación del circuito de apoyo a profesionales sanitarios confinados por COVID-19.

Cuestionario de evaluación del circuito de apoyo a profesionales sanitarios confinados por COVID-19.
Como profesional afectado por la COVID-19 ¿recibiste algún tipo de seguimiento telefónico por parte de este circuito mientras estabas confinado?
Respuesta SI: Inicia la encuesta Respuesta NO: Dirige a sugerencias
Valora de 0 (nada satisfactorio) a 3 (muy satisfactorio) los siguientes aspectos de las llamadas de seguimiento:
1) Frecuencia adecuada de las llamadas de seguimiento clínico.
2) Duración de las llamadas telefónicas.
3) Información recibida en las llamadas.
4) Resolución de las dudas planteadas durante las llamadas.
5) Apoyo recibido durante el seguimiento clínico.
Valora de 0 (nada útiles) a 3 (muy útiles) en qué medida las llamadas te han resultado útiles en los siguientes aspectos:
6) Bienestar emocional.
7) Gestiones relacionadas con aspectos laborales.
8) Relaciones familiares.
9) Manejo de síntomas.
Valora de 0 (nada satisfactorio) a 3 (muy satisfactorio) la tarea realizada por los siguientes colectivos implicados en el circuito:
10) Los profesionales que te han llamado para el seguimiento clínico.
11) Unidad de apoyo psicológico.
12) Unidad Básica de Prevención.
13) Circuito de realización de PCR.
14) Equipo directivo de tu centro.
15) Apoyo por parte de la Dirección del Área Sanitaria.
16) Coordinación entre los diferentes agentes implicados.
17) Facilidad para contactar con los profesionales que realizaban el seguimiento, en caso que haya sido necesario.
18) Si tuvieras que poner una nota al servicio de seguimiento en general. Puntúa del 1 al 10 (1 = muy malo, 10 = muy bueno).
19) Tienes alguna sugerencia de mejora o comentario.

### Instrumentos

Se elaboró un cuestionario ad hoc siguiendo las recomendaciones de Boynton y Greenhalgh^10^ para dotar de validez y fiabilidad los cuestionarios creados. La investigación se llevó a cabo a petición del Departamento de Calidad, por lo que el primer paso fue conocer qué temas se querían explorar. En segundo lugar, se decidió la metodología de recogida de datos más apropiada, por tratarse de cuantificación de medidas generales, y buscando la facilidad y rapidez para su cumplimentación se optó por un cuestionario de 17 preguntas con respuesta en escala numérica de 0 a 3, y una pregunta con respuesta de 1 a 10. Para ampliar los hallazgos se dio la posibilidad de expresarse en una pregunta abierta cuya respuesta se analizó con métodos cualitativos.

Se valoró como no recomendable la utilización de un instrumento de medida estandarizado por el contexto particular de pandemia que se vivió por primera vez en un ámbito sanitario determinado, para el cual cualquier cuestionario utilizado en otro contexto perdería su validez.

En relación con la apariencia del cuestionario se cuidaron los detalles relativos a fuente y tamaño de la letra y longitud de las preguntas para asegurar una tasa de respuesta elevada. Además, se realizaron 5 entrevistas cognitivas pretest con una muestra de potenciales participantes en el estudio para que evaluaran una primera versión del cuestionario mediante la técnica del think aloud^11^.

Se aseguró la validez del cuestionario a través de un procedimiento en el que se dieron instrucciones específicas a 6 jueces expertos a los que se les pidió que evaluaran la Interpretabilidad, Representatividad, Relevancia de los ítems puntuando de 1 a 4 cada uno de ellos, previa información sobre el propósito del cuestionario. También se alentó a los expertos a compartir comentarios sobre la redacción de los elementos y las opciones de respuesta, sugerencias de revisión, número y orden de los elementos, textos de instrucciones, elementos faltantes, titulares y diseño^12^. El Índice de Validez de Contenido para los Ítems finales (I-IVC) de respuesta cerrada, con 6 jueces expertos fue superior a 0,78 en todos los ítems. El α de Cronbach para los elementos de respuesta cerrada fue de 0,93.

### Análisis de Datos

Se realizó un análisis descriptivo de los datos cuantitativos que presentan las variables categóricas con distribución de frecuencias, y las variables cuantitativas con media, desviación estándar, moda y mediana. Para la comparación de medias se utilizó la prueba t de Student y ANOVA, y se consideró significativo todo valor de p < 0,05. Se utilizó el paquete SPSS, versión 25.

Mediante el programa ATLAS.ti 8.4, las respuestas a las preguntas abiertas se sometieron a un análisis de contenido temático sumativo, que involucra la codificación y el conteo de las respuestas con base en una matriz de códigos preestablecida e interpretación del significado latente de este contenido a partir de los contextos de uso y coocurrencias de los códigos^13^. Los análisis cuantitativo y cualitativo se llevaron a cabo de manera secuencial y explicativa, orientando el análisis cualitativo a la comprensión de los resultados cuantitativos.

### Consideraciones Éticas

La primera pregunta del cuestionario estuvo relacionada con el consentimiento informado, y si el participante lo aceptaba, podría continuar la encuesta. La participación fue voluntaria y se garantizó el anonimato desactivando la posibilidad de seguimiento de las respuestas, además de no solicitar datos que permitieran conocer la identidad del participante. El estudio fue aprobado por el Comité de Ética de Investigación Clínica del IDIAPJGol (Código 20/209-PCV).

## RESULTADOS

El enlace de la encuesta se envió a 1.247 personas, de las cuales 398 iniciaron la encuesta. En la primera pregunta 60 participantes contestaron no recordar haber recibido el apoyo que se estaba evaluando, por lo que la encuesta les dirigió a la pregunta abierta de comentarios y sugerencias, y 22 de ellos escribieron comentarios en ella. De los 338 participantes restantes que sí recordaban haber recibido el apoyo telefónico, 286 finalizaron la encuesta completa.

### Datos Sociodemográficos

La muestra tuvo mayor predominio femenino (81,8%). La edad media fue de 47 años con una desviación estándar de 10,4, la edad mínima 22 años y la máxima 65. Otras características sociodemográficas se describen en la Tabla 1.

**Tabla 1:** Características sociodemográficas.

n = 286	Sí	No
Pertenece a un grupo vulnerable	88 (30,8%)	198 (69,2%)
Convive con personas vulnerables	98 (34,3%)	188 (65,7%)
Convive con niños	166 (41,9%)	120 (58%)
Fue diagnosticado de COVID-19 por PCR	127 (44,4%)	159 (55,6%)
Conoce el origen del contagio	63 (46%)	74 (54%)

PCR: *polymerase chain reaction.*

El cuestionario fue contestado por enfermeras y médicos en el 63% y el resto por diferentes colectivos profesionales. La Tabla 2 muestra la distribución de los participantes según su profesión.

**Tabla 2:** Distribución de la muestra según su profesión (n = 286).

Profesión	n (%)
Enfermera/o	102 (35,7%)
Médica/o	80 (28%)
Gestión y servicios	49 (17,1%)
Técnico cuidados auxiliares de enfermería	24 (8,4%)
Trabajo social	7 (2,4%)
Odontología	6 (2,1%)
Técnico de gestión	4 (1,4%)
Comadrona	2 (0,7%)
Fisioterapeuta	1 (0,3%)
Otras profesiones	11 (3,8%)

### Resultados de la Evaluación Cuantitativa

La puntuación global media que obtuvo el circuito fue un 6,54, siendo la peor puntuación posible el 0 y la mejor posible un 10. La moda se situó en el valor 8, y la mediana en el 7. La Tabla 3 muestra la puntuación obtenida por cada uno de los ítems en una escala de 0 a 3, observando que las puntuaciones más altas las obtienen los ítems relacionados con los aspectos formales de las llamadas, y la puntuación más baja la obtiene la unidad de apoyo psicológico.

**Tabla 3:** Evaluación del circuito.

	Media^a^	DE	Moda^a^
Aspectos relacionados con las llamadas			
Frecuencia adecuada	2,11	0,89	3
Duración de las llamadas	2,37	0,73	3
Información recibida	2,16	0,86	3
Resolución de las dudas	2,11	0,88	3
Apoyo recibido	2,15	0,93	3
Utilidad de las llamadas			
Bienestar emocional	1,91	0,98	3
Gestiones laborales	1,88	1,02	3
Relaciones familiares	1,73	1,06	2
Manejo de síntomas	2,00	0,96	3
Valoración por colectivos			
Identificación	2,76	0,56	3
Profesionales de seguimiento	2,34	0,78	3
Unidad de Apoyo Psicológico	0,70	1,11	0
Unidad Básica de Prevención	1,64	1,21	3
Circuito de realización de PCR	1,66	1,18	3
Equipo directivo del centro	2,15	1,02	3
Apoyo de la dirección general	1,44	1,12	0
Coordinación y gestión			
Coordinación de colectivos	1,47	0,95	1
Facilidad de contacto	1,61	1,10	1/3

DE: desviación estándar.

^a^ 0 es la mínima valoración posible y 3 la máxima valoración posible.

Se realizó T test para comparar la media global obtenida según género, pertinencia a un grupo vulnerable, convivencia con personas vulnerables, tener hijos o no, resultado de la PCR, ser profesional de ciencias de la salud o no, y origen del contagio. No se encontraron diferencias significativas en ninguno de los casos. La Tabla 4 muestra los estadísticos de estas comparaciones de medias.

**Tabla 4:** Comparación por grupos de la puntuación global.

	Media	DE	t^a^	Sig
Género				
Hombre	6,88	2,49	1,11	0,27
Mujer	6,47	2,41		
Pertinencia a grupo vulnerable				
No	6,58	2,36	0,37	0,71
Sí	6,47	2,59		
Convivencia con personas vulnerables				
No	6,48	2,35	-0,64	0,52
Sí	6,67	2,57		
Hijos				
No	6,77	2,36	1,81	0,07
Sí	6,24	2,49		
Resultado PCR				
Negativo	6,50	2,50	-0,80	0,42
Positivo	6,74	2,34		
Profesión ciencias de la salud				
No	6,82	2,28	1,11	0,27
Sí	6,45	2,47		
Origen del contagio			F^b^	
Paciente	6,45	2,38	0,85	0,43
Comunitario	7,42	2,11		
Desconocido	6,74	2,38		

DE: desviación estándar.

^a^ Teste t de Student.

^b^ Teste F.

Se analizó con más detalle el resultado obtenido en la evaluación por la unidad de apoyo psicológico, ya que obtuvo una puntuación muy baja. Se realizaron análisis bivariados con todas las variables sociodemográficas y laborales, no encontrando ninguna asociación significativa.

### Resultados del Análisis de Contenido de las Respuestas a la Pregunta Abierta

Un total de 109 personas (27,4%) respondieron a la pregunta “Si tiene alguna sugerencia de mejora o comentario, lo puede escribir aquí” que se formuló a todos los participantes.

El análisis del contenido manifiesto de las respuestas, codificadas a partir de las tres dimensiones del enfoque bio-psico-social, mostró que la mayoría de los comentarios (n = 61) hicieron referencia a aspectos organizativos (dimensión social), seguidos de aquellos referidos a cuestiones psicológicas (dimensión psicológica; n = 22) y, finalmente, a temas relativos al manejo de síntomas (dimensión biológica; n = 10).

#### Dimensión social

A pesar del enfoque deductivo del análisis, categorizando los datos en las tres dimensiones de la orientación biopsicosocial, el análisis del contexto en el que se hacían los comentarios y sus coocurrencias mostró cómo se entrelazaban comentarios referidos a la vivencia más psicológica (“me sentí muy sola”) y del proceso biológico de la enfermedad (“estuve 11 días con fiebre”), con explicaciones de acciones u omisiones concretas a nivel de la gestión socio-organizativa de la situación (“reclamar la realización de la PCR”):

*Durante mi enfermedad me sentí muy sola, tuve que llamar yo para reclamar la realización de la PCR, tanto al inicio del cuadro como la de preincorporación. Estuve 11 días con fiebre y no recibí ni una llamada de seguimiento, fui yo quien decidió hacerme una Rx de Tórax, gestionarla, revisarla y ver que había una pneumonía, hablar con un médico internista, quien me prescribió medicación. No me sentí apoyada en ningún momento. Me gustaría que no se volviera a repetir una vivencia como esta. (43:2)*

Como en este comentario, los profesionales ubicaban su experiencia del aislamiento en un lugar u otro del continuo acompañamiento-soledad como efecto de una combinación de diversas prácticas concretas, vinculadas muchas veces a la resolución de aspectos formales, que tuvieron lugar o no durante el seguimiento (por ejemplo, la realización de pruebas diagnósticas).

#### Dimensión psicológica

*En la primera parte del proceso vivido, mi percepción fue de abandono total y la no realización de PCR hasta que ingresé en el hospital con una pneumonía después de 15 días de iniciar mi confinamiento. Una vez dado de alta, sí, me sentí muy reconfortado por las llamadas de la Dra. M. Me sirvió mucho de apoyo psicológico y emocional. Le estoy muy agradecido. A la UPR, un cero. (88:3)*

En este extracto se constata la vinculación entre la percepción de soledad y algunas de las fragilidades del circuito, especialmente en su inicio (la realización de PCR y el papel de la Unidad Básica de Prevención –en esta cita “UPR”–). Sin embargo, también se contrapone a esta soledad el papel del apoyo psicológico. La “Dra. M” pertenece a la Unidad de Apoyo Psicológico, que se activaba en casos puntuales. Hay pocas referencias explícitas al “apoyo psicológico” (n = 5) en el conjunto de respuestas abiertas y todas, excepto esta muestra de agradecimiento, son peticiones expresas de ampliar o integrar (n = 4) este tipo de servicio.

Por consiguiente, parece pertinente establecer una distinción entre las valoraciones de la unidad de apoyo psicológico y la percepción de sentirse acompañada emocionalmente como consecuencia de ser atendido en el marco de este circuito de seguimiento.

#### Dimensión biológica

Tal y como se apunta más arriba, el valor del circuito en tanto “apoyo emocional” no se puede atribuir exclusivamente al desempeño de la Unidad de Apoyo Psicológico, sino parece emerger más bien como efecto de una combinación de diversas prácticas concretas que tuvieron lugar o no durante el seguimiento, incluyendo aquellas referidas al manejo de síntomas y “autocontrol de la enfermedad” como se detalla en el siguiente extracto.

*Valoro imprescindible el acompañamiento, por el soporte sobre los síntomas y las incertidumbres, sobretodo al principio de la pandemia, que es cuando yo me infecté. De mi proceso, además, destacaría el apoyo emocional recibido, no lo esperaba y lo encontré y lo agradecí. Somos “pacientes” pero a la vez profesionales de la salud, hecho que ayuda al autocontrol de la enfermedad pero, a la vez, también a anticiparse. (106:4)*

## DISCUSIÓN

Los indicadores de proceso y resultado de los servicios sanitarios tienen un papel crucial en la satisfacción de los pacientes^14^. En esta línea, la intervención implementada estaría en la línea de un Walkround, práctica común asociada con la seguridad del paciente, en que la retroalimentación es una intervención potente^15^.

Esta investigación pone de manifiesto la capacidad de respuesta del sistema sanitario ante una crisis provocada por la pandemia de la COVID-19 que colocó al sistema sanitario en una situación de estrés sin precedentes, y que se tuvo que dar respuesta a los profesionales sanitarios en su rol de pacientes. Los profesionales afectados valoraron la respuesta como satisfactoria al puntuar la intervención de manera global con un 6.54.

La evaluación realizada se diseñó meticulosamente asegurando que las preguntas no contuvieran sesgos de interpretación y que los contenidos fueran los que realmente se pretendieron evaluar. El análisis de los datos, en el que ninguna variable fue predictora de los resultados, pone de manifiesto que los participantes respondieron al cuestionario dejando de lado las situaciones sociales y emocionales que les pudieran afectar. Por último, las características de los participantes fueron muy similares a las del total de confinados, como muestran los resultados de edad media de 47 años (45 en el total de profesionales confinados) y el elevado porcentaje de mujeres el 81,8% (78,8% en el total de profesionales confinados)^9^.

Los aspectos formales de las llamadas fueron evaluados de manera positiva, situándose en la parte alta una categorización de las respuestas.

Un aspecto importante en la implementación del circuito fue su vocación biopsicosocial, que se puede evaluar como conseguida, ya que las puntuaciones de la sección “utilidad de las llamadas” se encuentran en la parte alta. Los resultados cualitativos demuestran que, en efecto, existen diferentes concepciones de la utilidad que este tipo de seguimiento debe tener y que, cuando no se aprecia esta triple vertiente, el circuito pierde valor.

Se pueden plantear diferentes hipótesis para explicar este efecto a la luz de los resultados y la literatura relacionada. Por ejemplo, si la preponderancia se encuentra en los aspectos laborales, se puede balancear la percepción hacia una intervención de presión y control de recursos humanos, y no de apoyo^16^. Por otro lado, un circuito demasiado centrado en los aspectos clínicos puede no ser bien recibido por profesionales sanitarios a los que se les supone un cierto bagaje en este campo. Por último, de manera análoga, un seguimiento estrictamente psicológico puede ser rechazado por un colectivo en el que, paradójicamente, existe una considerable estigmatización de los problemas de salud mental^17^. Considerando que la literatura evidencia que una proporción considerable de trabajadores sanitarios han experimentado trastornos del sueño y del estado de ánimo durante la pandemia^18^, que los daños morales y el desarrollo de enfermedades mentales son riesgos reales cuando se trabaja en escenarios sin precedentes como este, el presente estudio incide en que se deberá trabajar de manera proactiva para evitar que las consecuencias psicológicas de la pandemia en el personal sanitario sean dramáticas^19^.

La evaluación de los diferentes colectivos que participaron en el circuito arrojó puntuaciones heterogéneas, y entre ellas destacó la muy baja puntuación que obtuvo la Unidad de Apoyo Psicológico del que el análisis cuantitativo no pudo encontrar predictores que lo explicaran. Sin embargo, los resultados del análisis de las respuestas abiertas permiten elaborar algunas explicaciones plausibles. Por un lado, el trabajo de la Unidad de Apoyo Psicológico no se valora negativamente, sino más bien se critica que no tuviera más presencia. Además, se desprende de los datos que la percepción de apoyo o acompañamiento emocional va más allá del trabajo de esta Unidad y emerge como resultado de una suma de pequeñas acciones, muchas veces vinculadas con la gestión, la información y la coordinación, facilitando una atención personalizada y más empática.

En este sentido, la puntuación obtenida en la coordinación de los diferentes colectivos implicados se encuentra por debajo del punto medio. Además, tal y como expresan los comentarios de los participantes, la situación sobrevenida les hace ser comprensivos hacia esta falta de coordinación y la dificultad de la implementación de cualquier iniciativa, lo cual les puede haber hecho ser benevolentes en sus puntuaciones.

Las evidencias obtenidas permiten elaborar una explicación que convierte lo desconocido en conocido, construida a partir de un proceso enfocado en opciones, experiencia y errores, áreas que intervienen en el proceso de reconocimiento de problemas en sus primeras etapas de desarrollo, el primer principio de las organizaciones de alta fiabilidad^20^.

Ante la evidencia de posibles afectaciones a la salud mental de los profesionales sanitarios durante la pandemia, diversas instituciones y organismos pusieron en marcha estrategias para abordar este tipo de problemáticas^21^. En España, se implementó y evaluó la eficacia de una intervención basada en dispositivos móviles orientada a prevenir y mitigar los problemas de salud mental más comunes en esta situación^22^. Los resultados de esta evaluación en concreto no mostraron un impacto significativo de la intervención, por lo que resulta especialmente pertinente complementar este tipo de evidencia con las valoraciones que hacen los colectivos a las que van dirigidas.

Existen algunas limitaciones en este estudio debido a la baja tasa de respuesta, y aunque se desconocen los motivos para la no participación del resto de las personas invitadas a participar, lo que podría explicarse por el lapso de tiempo entre el período de confinamiento y la realización de la encuesta. Sin embargo, la heterogeneidad de las características de la muestra y de los comentarios de la pregunta abierta no parecen mostrar un sesgo de selección.

En conclusión, se ha evaluado la implementación de un circuito biopsicosocial de apoyo a profesionales sanitarios de atención primaria afectados por la COVID-19 que se puso en marcha en los primeros momentos de la pandemia. Los profesionales han percibido el circuito como útil, ya que el valor número de puntuaciones se encuentra en los valores altos de la escala, sin embargo, se reconocen algunos aspectos de mejora. Se ha encontrado variabilidad en las respuestas derivada de las diferentes expectativas que pueden tener los profesionales por las diferentes adaptaciones que se fueron llevando a cabo a medida que aumentaba el conocimiento del virus y por la comprensión de la dificultad del escenario en el que se estaba trabajando.
